# Efficacy of a Virtual 3D Simulation–Based Digital Training Module for Building Dental Technology Students’ Long-Term Competency in Removable Partial Denture Design: Prospective Cohort Study

**DOI:** 10.2196/46789

**Published:** 2024-04-05

**Authors:** KeXin Liu, YaQian Xu, ChaoYi Ma, Na Yu, FaBing Tan, Yi Li, YaXin Bai, XiaoMing Fu, JiaWu Wan, DongQi Fan, HuBin Yin, MeiXi Chen, HongJi Chen, Lin Jiang, JinLin Song, Ping Ji, XiaoHan Zhao, MengWei Pang

**Affiliations:** 1 College of Stomatology, Chongqing Medical University, Chongqing, China; 2 Chongqing Key Laboratory of Oral Diseases and Biomedical Sciences, Chongqing, China; 3 Chongqing Municipal Key Laboratory of Oral Biomedical Engineering of Higher Education, Chongqing, China; 4 Beijing Unidraw Virtual Reality Technology Research Institute Co, Ltd, Beijing, China; 5 State Key Laboratory of Virtual Reality Technology and Systems, BeiHang University, Beijing, China

**Keywords:** removable partial denture, RPD, virtual simulation, dental technology, computer-aided design, CAD, clinical practice, efficacy, cohort study, digital training, training, dentistry, treatment, design, virtual, assessment

## Abstract

**Background:**

Removable partial denture (RPD) design is crucial to long-term success in dental treatment, but shortcomings in RPD design training and competency acquisition among dental students have persisted for decades. Digital production is increasing in prevalence in stomatology, and a digital RPD (D-RPD) module, under the framework of the certified Objective Manipulative Skill Examination of Dental Technicians (OMEDT) system reported in our previous work, may improve on existing RPD training models for students.

**Objective:**

We aimed to determine the efficacy of a virtual 3D simulation–based progressive digital training module for RPD design compared to traditional training.

**Methods:**

We developed a prospective cohort study including dental technology students at the Stomatology College of Chongqing Medical University. Cohort 1 received traditional RPD design training (7 wk). Cohort 2 received D-RPD module training based on text and 2D sketches (7 wk). Cohort 3 received D-RPD module pilot training based on text and 2D sketches (4 wk) and continued to receive training based on 3D virtual casts of real patients (3 wk). RPD design tests based on virtual casts were conducted at 1 month and 1 year after training. We collected RPD design scores and the time spent to perform each assessment.

**Results:**

We collected the RPD design scores and the time spent to perform each assessment at 1 month and 1 year after training. The study recruited 109 students, including 58 (53.2%) female and 51 male (56.8%) students. Cohort 1 scored the lowest and cohort 3 scored the highest in both tests (cohorts 1-3 at 1 mo: mean score 65.8, SD 21.5; mean score 81.9, SD 6.88; and mean score 85.3, SD 8.55, respectively; *P*<.001; cohorts 1-3 at 1 y: mean score 60.3, SD 16.7; mean score 75.5, SD 3.90; and mean score 90.9, SD 4.3, respectively; *P*<.001). The difference between cohorts in the time spent was not statistically significant at 1 month (cohorts 1-3: mean 2407.8, SD 1370.3 s; mean 1835.0, SD 1329.2 s; and mean 1790.3, SD 1195.5 s, respectively; *P*=.06) but was statistically significant at 1 year (cohorts 1-3: mean 2049.16, SD 1099.0 s; mean 1857.33, SD 587.39 s; and mean 2524.3, SD 566.37 s, respectively; *P*<.001). Intracohort comparisons indicated that the differences in scores at 1 month and 1 year were not statistically significant for cohort 1 (95% CI –2.1 to 13.0; *P*=.16), while cohort 3 obtained significantly higher scores 1 year later (95% CI 2.5-8.7; *P*=.001), and cohort 2 obtained significantly lower scores 1 year later (95% CI –8.8 to –3.9; *P*<.001).

**Conclusions:**

Cohort 3 obtained the highest score at both time points with retention of competency at 1 year, indicating that progressive D-RPD training including virtual 3D simulation facilitated improved competency in RPD design. The adoption of D-RPD training may benefit learning outcomes.

## Introduction

The partially edentulous population is increasing because of increased life expectancy and an aging population [1]. Removable partial dentures (RPDs) possess the advantages of cost-effectiveness and needing a less invasive procedure compared to fixed and implant-retained restorations; thus, RPDs remain an attractive treatment option for partially edentulous patients [2].

The design of RPDs is a crucial technical step that greatly impacts the long-term success of dental treatment and warrants high standards due to the complex structure and variation in the oral morphology of individual patients [3,4]. Poor RPD design can exacerbate plaque retention, leading to gingivitis, periodontitis, and other oral diseases [5]. RPD design has traditionally been a complex subject to teach and learn [6]. Unfortunately, shortcomings in RPD design training and competency acquisition among dental students have persisted for decades [7]. The lack of student supervision by qualified instructors and progressive training patterns, as well as the absence of practice on real patients, have been found to be the main factors limiting successful training in RPD design [8,9]. The lack of competency in RPD design can hamper clinical practice among dentists, often leading to the assignment of the task to dental technicians. Dental technicians, however, lack direct observation of the oral soft and hard tissues of the patients. This factor can limit the quality of prosthesis design and can cause patient discomfort, resulting in additional repairs and medical disputes [10].

Using a pencil-drawn design of the RPD framework on a physical cast or a paper prescription has always been the classic approach for teaching RPD design in most dental schools [11]. However, this classic approach is marked by several constraints. The cumbersome processes used where teachers collect, rate, and hand out paper prescriptions can result in communication gaps and potential wastage of time [11]. The COVID-19 pandemic has further limited the availability of real, patient-based physical casts, thus eroding practice time for RPD design on patient models [12]. Although advances in the dental laboratory digital workflow facilitate the use of computer-aided design (CAD) and computer-aided manufacturing (CAM) in the fabrication of RPDs and communication between dentists and dental technicians [13], multiple surveys confirm that CAD/CAM RPD design courses continue to present significant barriers to widespread adoption in dental education settings due to the cost, lack of faculty, and lack of time available within the curriculum. Moreover, the education editions of commercial CAD software programs for dental laboratories remain expensive and require instructors proficient in CAD/CAM technology to facilitate teaching. Furthermore, the learning curve to master the skills of using commercial CAD software is steep and requires a long time commitment, which presents a problem in undergraduate dental education settings.

In our previous work, we reported a digital RPD (D-RPD) module under the framework of the certified Objective Manipulative Skill Examination of Dental Technicians (OMEDT) system, which is a free web-based application for computer-aided drawing and 2D sketch–based RPD design training for dental and dental technology students [14]. This prospective cohort study aimed to report a significant update to the D-RPD module and to further explore the optimal design of the D-RPD module for teaching. We specifically asked the following questions: (1) How can a progressive approach using case-based virtual 3D simulation be incorporated in a D-RPD design training module to better prepare students for the needs of practice? (2) What is the efficacy of digital training approaches in RPD design compared to traditional training? (3) Does a virtual 3D simulation–based progressive digital training module benefit long-term RPD design competency acquisition and retention?

## Methods

### Development of a Progressive D-RPD Module Incorporating Case-Based Virtual 3D Simulation

The virtual 3D simulation was based on casts from actual patients. In order to construct patient-based virtual casts, a desktop portable application, showModels, has been developed with the Unity engine and C++ version 11 and C# version 4.0. All clinical cases used in the RPD design training were collected from the Dental Technology Laboratory of the Stomatology Hospital of Chongqing Medical University. Virtual casts were constructed from physical plaster casts of clinical patients using LabScanner (E4; 3shape) and saved in the stereolithography file format using Format Converter (Autodesk; Delcam Exchange) to remove possible surface texture indicators. Since any prepared rest seats on a patient’s physical casts may provide hints for RPD design, such rest seats on the virtual cast were filled using 3D reverse software (Geomagic Wrap; 3D System). The resultant virtual casts of real patients may be rotated or zoomed in and out to view the cast details, and the user may specify whether to display the maxillary or the mandibular cast (Multimedia Appendix 1).

### Participants and Recruitment

Eligible participants (junior students majoring in dental technology) were recruited at the Stomatology College of Chongqing Medical University. The RPD design theory curriculum in dental technology was organized by the Stomatology Hospital of Chongqing Medical University. All participants provided signed informed consent. The prospective cohort study began in September 2020 and ended in September 2022.

### Ethical Considerations

The Research Ethics Committee of the Affiliated Hospital of Stomatology, Chongqing Medical University, approved this study protocol (COHS-REC-2022; LSNo. 096). Data reporting followed the Strengthening the Reporting of Observational Studies in Epidemiology (STROBE) guidelines for cohort studies.

### Intervention Design

**Figure 1.**
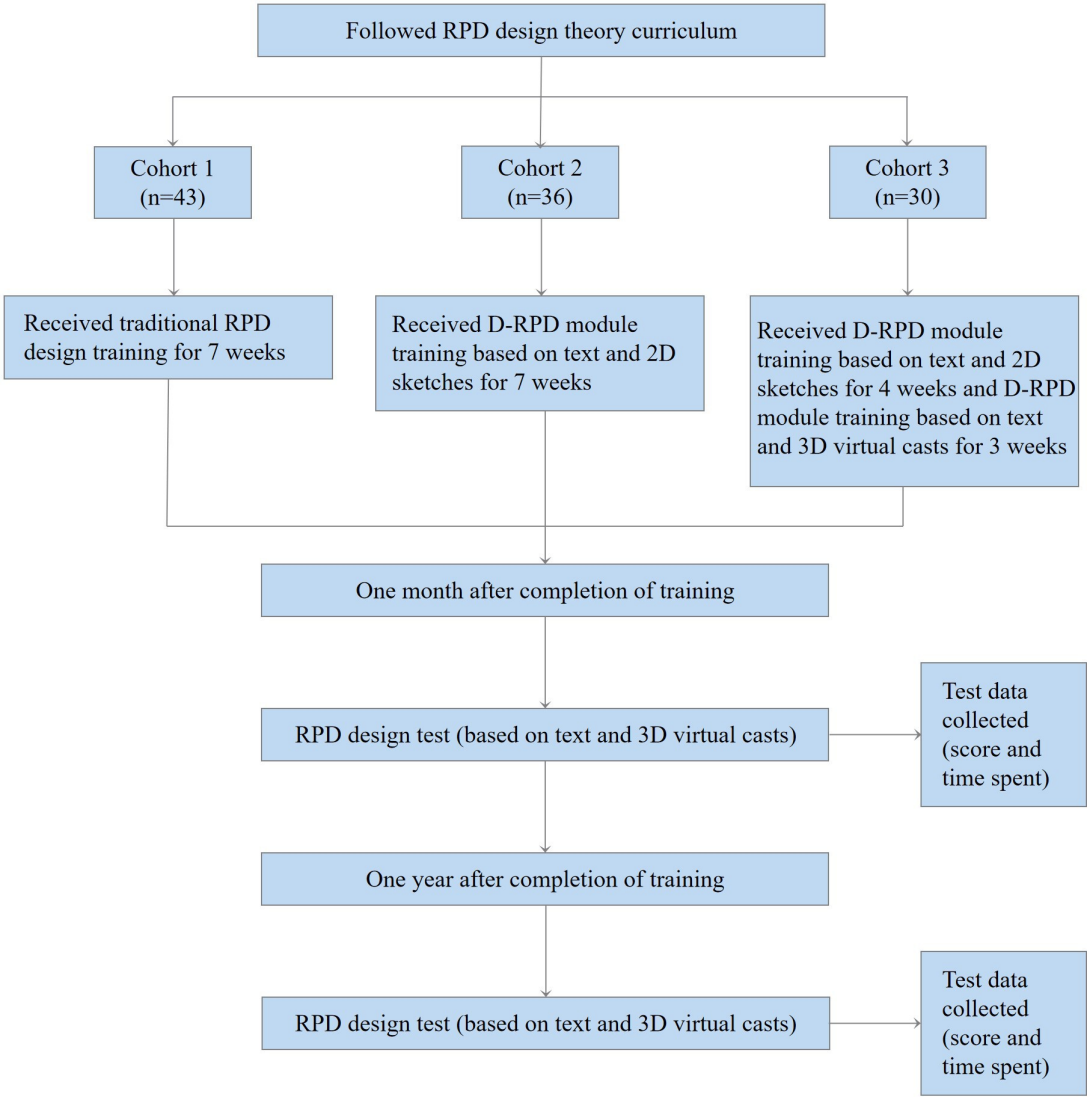
The flowchart of this prospective cohort study. D-RPD: digital removable partial denture; RPD: removable partial denture.

The study protocol and the participant flow diagram are depicted in Figure 1. A description of the training methods implemented is presented in Multimedia Appendix 2. In brief, after following the same RPD design theory curriculum, all participants were divided into 3 cohorts. Cohort 1 included 43 participants who received traditional RPD design training for 7 weeks. They received an RPD design task from the principal investigator each Monday, completed the RPD design using a paper prescription and red and blue pencil, and submitted it by Sunday. Cohort 2 included 36 participants who received D-RPD module training based on literal descriptions and 2D sketches for 7 weeks. They received literal case descriptions and 2D sketches for uniformly depicting missing teeth issued by the principal investigator in the D-RPD module every Monday, drew RPD designs using the D-RPD module, and submitted designs by Sunday. Cohort 3 included 30 participants who received D-RPD module training based on a literal description and 2D sketches for 4 weeks and continued to receive progressive instruction with the updated D-RPD module training based on the virtual casts of real patients for 3 weeks. The 7 RPD design tasks received by the 3 cohorts were all the same, and the types of dentition defects covered Kennedy classes I, II, III, and IV, with only some differences in presentation form. We set 1 month as the retention interval to avoid temporary effects from practice [15]. At 1 month and at 1 year after the training, RPD design tests using 3D virtual casts were administered using the updated D-RPD module and carried out for all of the cohorts. During the retention interval, participants’ D-RPD module accounts were blocked to prevent participants from using the module for additional training. Within 1 year of completing their training, participants start a uniform dental laboratory internship, and the internship outline has uniform requirements for the design of RPDs with the same workload. For cohorts 1 and 2, a separate D-RPD module introductory session was held prior to the testing to ensure that the cohort could successfully complete the RPD design task using the updated D-RPD module.

### Recruitment of the Expert Panel and Development of the Scoring Rubrics

The principal investigator recruited an expert panel to develop the scoring rubrics [16] (Table 1), and the exercises of all 3 cohorts were rated accordingly. The expert panel consisted of a dental technician experienced in the field of RPD manufacturing and a clinical prosthodontist recruited from the Stomatology Hospital of Chongqing Medical University. The expert panel was blinded to the cohort assignments, had not participated in the teaching of the participants, and did not know about the participants’ major or nature of the intervention.

**Table 1.** The scoring rubric used to assess the removable partial denture design test task.

Scoring component	Met clinically acceptable criteria	Needs improvement	Clinically unacceptable
Case observation (20 points)	The missing tooth position was identified accurately and marked correctly on the drawing (20 points).	N/A^a^	The missing tooth position was identified inaccurately, marked incorrectly on the drawing, or both (0 points).
Design choices (40 points)	Design choices are ideal for the case (28-40 points).	Design choices have some flaws but are adequate (15-27 points). Examples include the following: no missing component; indirect retainer present but not in the optimal position; design choices do not violate biological principles; clasp choice adequate but not optimal for the case; inappropriate choice or extension of major connector; justified use of clasps/rests but excessive framework components.	Any missing component or inappropriate design for the case (0-14 points). Examples include the following: missing indirect retainer in a case requiring one; missing reciprocation; clasp choice inappropriate for situation; design choices violate biological principles; excessive and unjustified use of clasps/rests.
Drawing (20 points)	Drawing is ideal. Metal components are painted in blue and resin bases are in red (14-20 points).	Drawing has some flaws but is adequate (7-13 points). Examples include the following: components are represented by corresponding colors; minor inadequacy or inconsistency of spacing between components; components are occasionally not connected; the finish line is not drawn.	Drawing has major flaws (0-6 points). Examples include the following: components are not represented by corresponding colors; major inadequacy or inconsistency of spacing between components; component positioning significantly off optimal position; any component position that violates biomechanical design principles; components are frequently not connected; the finish line is not drawn.
Consistency with task description (10 points)	Exactly as described in the task description (8-10 points). Criteria include the following: clearly presents the requirements implied in the description, and the design is well aligned with the corresponding description; gives consideration to both aesthetics and function.	Some deviation from the task description, but it is acceptable (5-7 points). Examples include the following: conventional design carried out without addressing case-specific modifying factors or requirements listed in the task description; only function considered, consideration for aesthetics lacking.	Serious violation or deviation from the task description (0-4 points). Examples include the following: the design does not match the task description; lack of aesthetic and functional considerations.
Neatness and accuracy in presentation (10 points)	Neat and accurate, no inconsistencies between the table and drawing (8-10 points).	Some inaccuracy and neatness flaws, but it is adequate (5-7 points). Examples include the following: minor erasures; minor neatness issues but still legible.	Major inaccuracy and neatness flaws (0-4 points). Examples include the following: missing information; major neatness issues; writing not legible; any inconsistencies between the table and drawing.

a N/A: not applicable.

### Data Collection

The main metrics collected were the time(s) to complete the RPD design exercise and the RPD design score (100 points) based on the scoring rubrics by the expert panel.

### Statistical Analysis

Scores (ie, total points for each assessment) and time (ie, seconds needed to perform each assessment) were summarized descriptively as means and SDs, coefficients of variation (defined as the ratio of the SD to the mean), and IQRs [17]. Due to the small sample size, normality was tested using the Shapiro-Wilk test, and the homogeneity of variance was tested using the *F* test [18]. The results showed that the time and score data had a skewed distribution and heterogeneity of variance. Therefore, the nonparametric method was used to compare the data sets in this study. Since the Kruskal-Wallis test is widely used to determine whether 3 or more independent data sets are different on some variable of interest [19], it was used to compare the cohorts at the same time point (1 mo or 1 y later), using the 3 data sets in each analysis process. When the value of the Kruskal-Wallis statistic is calculated as statistically significant, it indicates that at least 1 of the compared groups is different from the others. Therefore, we chose the Bonferroni method for further analysis with pairwise multiple comparisons to locate the source of significance. As for in-cohort comparisons at different time points, the Wilcoxon matched-pairs test is a frequently used nonparametric test for paired data, especially for nonnormal data and categorical data, such as was present in this cohort study. Hypothesis tests were 2 sided with a significance threshold of *P*=.05. At the same time, when multiple sets of data are being processed and compared simultaneously, there is increased risk of a type I error, so to identify significant correlations, threshold levels of significance for correlation coefficients were adjusted for multiple comparisons; we used a set of κ correlation coefficients with Bonferroni correction to strictly control the occurrence of false positives (after Bonferroni correction, we used a significance threshold of *P*=.016) [20]. Statistical analysis was performed using SPSS (version 26.0; IBM Corp).

## Results

This cohort study included 109 participants: 58 (53.2%) women and 51 (56.8%) men, with a mean age at the beginning the study in September 2020 of 22.5 (SD 0.7) years (Table 2). All 3 cohorts completed the experiment.

**Table 2.** Baseline characteristics of the participants in this cohort study. *P* values were estimated using the Kruskal-Wallis test.

Characteristics		Participants overall (n=109)	Cohort 1 (n=43)	Cohort 2 (n=36)	Cohort 3 (n=30)	*P* value
**Gender, n (%)**						.57
	Female	58 (53.2)	26 (60.5)	18 (50)	14 (46.7)	
	Male	51 (46.8)	17 (39.5)	18 (50)	16 (53.3)	
	Other	0 (0)	0 (0)	0 (0)	0 (0)	
Age (years), mean (SD)		22.5 (0.7)	22.3 (0.7)	22.6 (0.7)	22.5 (0.7)	.40

### Intercohort Comparison of Performance

#### Scores

**Figure 2.**
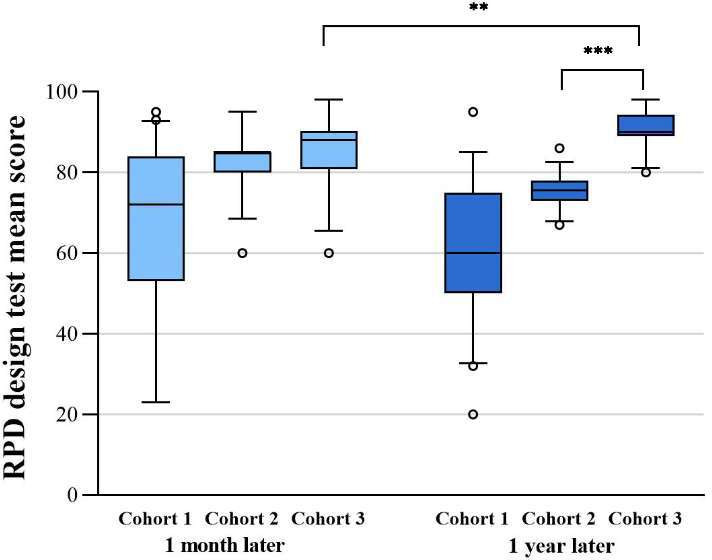
Intercohort comparison and intracohort comparison of the score of the removal partial denture (RPD) design test. **P≤.01, ***P≤.001.

The scores of cohorts 1, 2, and 3 after 1 month showed a statistically significant difference (mean 65.8, SD 21.5; mean 81.9, SD 6.9; and mean 85.3, SD 8.6, respectively; *P*<.001). Pairwise comparisons showed that the mean score of cohort 1 was 16.1 points less than the mean score of cohort 2 (95% CI –23.0 to –9.0; *P*=.03) and 19.5 points less than that of cohort 3 (95% CI –26.7 to –12.2; *P*<.001), whereas the difference in scores between cohorts 2 and 3 was not statistically significant (95% CI –7.3 to 0.48; *P*=.29). At testing after 1 year, the scores of cohorts 1, 2, and 3 showed a statistically significant difference (mean 60.3, SD 16.7; mean 75.5, SD 3.9; and mean 90.9, SD 4.3, respectively; *P*<.001). Pairwise comparisons showed that the mean score of cohort 1 was 15.2 points less than that of cohort 2, but this was not significantly different (95% CI –20.5 to –9.9; *P*=.06). Meanwhile, the mean score for cohort 3 was 30.6 points higher than that of cohort 1 (95% CI –36.0 to –25.2; *P*<.001), and the mean score of cohort 3 was 15.4 points higher than that of cohort 2 (95% CI –17.4 to –17.3; *P*<.001); both represented a highly significant difference (Table 3 and Figure 2).

**Table 3.** Intercohort comparison of the score and time spent on removal partial denture (RPD) design tests conducted after 1 month and after 1 year; intracohort comparisons of the score and time spent between the 1 month and 1 year time points.

		After 1 month							After 1 year							
		Mean (SD)	Coefficient of variation	Quartile 1 (IQR; range)	*P* value^a^	Difference^b^ (95% CI; *P* value^c^)	Difference^d^ (95% CI; *P* value)^e^	Difference^f^ (95% CI; *P* value^g^)	Mean (SD)	Coefficient of variation	Quartile 1 (IQR; range)	*P* value^a^	Difference^b^ (95% CI; *P* value^c^)	Difference^d^ (95% CI; *P* value^e^)	Difference^f^ (95% CI; *P* value^g^)	*P* value^h^
**RPD design test score**					<.001	–16.0 (–23.0 to –9.0; .003)	–19.5 (–26.7 to –12.2; <.001)	–3.4 (–7.3 to 0.48; .29)				<.001	–15.2 (–20.5 to –9.9; .006)	–30.6 (–36.0 to –25.2; <.001)	–17.4 (–17.4 to –17.3; <.001)	
	Cohort 1	65.8 (21.5)	0.33	53.0 (31.00; 23-95)					60.3 (16.7)	0.28	50.0 (25.00;20-95)					.16^i^
	Cohort 2	81.9 (6.9)	0.08	80.0 (5.00; 60-95)					75.5 (3.9)	0.05	73.0 (5.00;86-67)					<.001^i^
	Cohort 3	85.3 (8.6)	0.10	80.8 (9.50; 60-98)					90.9 (4.3)		89.0 (5.25;80-98)					.001^j^
**Time spent, s**					.06	572.9 (–33.8 to 1179.5; >.99)	567.5 (14.4 to 1120.6; >.99)	44.65 (–576.7 to 666.0; >.99)				<.001	191.8 (–195.7 to 579.3; >.99)	–475.2 (–868.3 to –82.0; <.001)	–667.0 (–951.6 to –382.4; .004)	
	Cohort 1	2407.8 (1370.3)	0.57	1088.0 (2132.00; 293-5286)					2049.2 (1099.0)	0.54	1256.0 (1393.00;364-4623)					.10^i^
	Cohort 2	1835.0 (1329.2)	0.72	938.0 (1317.75; 65-5950)					1857.3 (587.4)	0.32	1617.5 (567.25;593-3224)					.31^j^
	Cohort 3	1790.3 (1195.5)	0.67	901.5 (1284.50; 553-4614)					2524.3 (566.4)	0.23	2380.8 (535.25;837-3276)					.003^j^

a Kruskal-Wallis *H* test for differences in the score or time spent among the 3 cohorts.

b Cohort 1 vs cohort 2.

c Kruskal-Wallis *H* test for differences in the score or time spent between cohort 1 and cohort 2.

d Cohort 1 vs cohort 3.

e Kruskal-Wallis *H* test for differences in the score or time spent between cohort 1 and cohort 3.

f Cohort 2 vs cohort 3.

g Kruskal-Wallis *H* test for differences in the score or time spent between cohort 2 and cohort 3.

h *P* value for differences in both the score and time spent between the 1 month and 1 year time points.

i Paired 2-tailed *t* test for differences.

j Wilcoxon matched-pairs test for differences.

#### Time Spent

**Figure 3.**
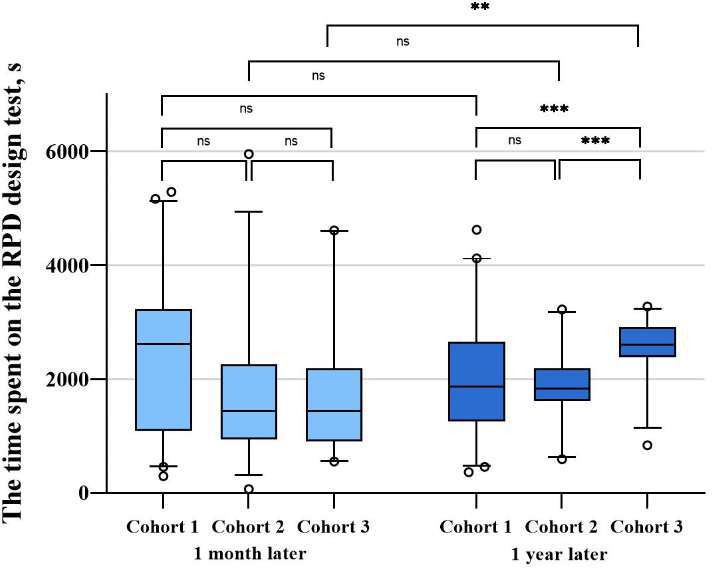
Intercohort comparison and intracohort comparison of the time spent on the removal partial denture (RPD) design test. ns: not significant. **P≤.01, ***P≤.001.

No significant difference was noted in the time spent by the 3 cohorts on the test after 1 month (cohorts 1-3: mean 2407.8, SD 1370.3 s; mean 1835.0, SD 1329.2 s; and mean 1790.3, SD 1195.5 s, respectively; *P*=.06). Pairwise comparisons also did not show any significant differences (cohorts 1-3: 95% CI –33.8 to 1179.5; *P*>.99; 95% CI 14.4-1120.6; *P*>.99; 95% CI –576.7 to 666.0; *P*>.99, respectively). However, the mean time spent on the test after 1 year did show a statistically significant difference between the cohorts (cohorts 1-3: mean 2049.2, SD 1099.0; mean 1857.3, SD 587.4; and mean 2524.3, SD 566.4, respectively; *P*<.001). Pairwise comparisons showed that the mean time spent by cohort 1 was 745.1 seconds shorter than that by cohort 3 (95% CI –868.3 to –82.0; *P*<.001), and the mean time spent by cohort 2 was 667.0 seconds shorter than that by cohort 3 (95% CI –951.6 to –382.4; *P*=.004); both represent a statistically significant difference, while the difference between cohorts 1 and 2 was not statistically significant (95% CI –195.7 to 579.3; *P*>.99) (Table 3 and Figure 3).

### Intracohort Comparison of Performance at Different Time Points

#### Scores

The difference in scores between the tests conducted 1 month and 1 year later for cohort 1 was not statistically significant (95% CI –2.1 to 13.0; *P*=.16). For cohort 2, the mean score obtained on the test conducted 1 month later was 6.4 points higher than that obtained 1 year later (95% CI 3.9-8.8; *P*<.001). For cohort 3, the mean score obtained on the test conducted 1 month later was 5.6 points less than that obtained on the test conducted 1 year later (95% CI –8.7 to –2.5; *P*=.001) (Table 3 and Figure 2).

#### Time Spent

The time spent by cohorts 1 and 2 on the tests conducted 1 month and 1 year later did not differ significantly (cohort 1: 95% CI –77.5 to 794.9; *P*=.10; cohort 2: 95% CI –372.5 to 327.9; *P*=.31). However, a significant difference was observed for cohort 3, where the time spent on the test conducted 1 month later was 734.0 seconds shorter than that conducted 1 year later (95% CI –1149.9 to –318.0; *P*=.003) (Table 3 and Figure 3).

## Discussion

### Principal Findings

Historically, the process of learning RPD design is a potentially difficult part of dental education [21]. It requires that dental students first acquire a knowledge base and then use critical thinking skills based on evidence to apply that knowledge to a wide variety of clinical patient care situations. This characteristic suggests that a case-based learning mode is the most appropriate approach for RPD design learning. Case-based learning requires the use of real patient cases and scenarios to reflect realistic patient care situations, and students are asked to draw from their established foundational knowledge to make decisions about problems they may encounter in practice [22]. Previous studies have confirmed the effectiveness of case-based learning in RPD design learning [23]. These studies have typically used text and 2D sketches to describe structured clinical cases, but enhanced digital techniques are gradually being applied to transition from simple presentation documents to computer-aided teaching [11,24-27]. Some studies have further developed decision support systems for RPD design based on clinical case libraries to help trainee dentists complete RPD design by providing cases with similar task requirements [28,29]. More recently, 3D virtual casts and CAD software have been introduced to align with clinical cases and currently prevalent dental laboratory digital workflows [30,31]. Nevertheless, several challenges limit the application of these findings. First, many studies have only addressed students in clinical dental programs, ignoring the dental technology student populace, who, as future dental technicians, are key stakeholders for any RPD design education. Second, in assessing the validity of a training program, most studies have investigated short-term effectiveness without considering the effect on long-term retention of skills, which is the most important for translation to future practice. Our research approach fills these gaps.

When investigating relatively permanent changes in learning, the experimental design needs to incorporate a retention interval, which refers to a period without further practice. Following this interval, assessments can be conducted to evaluate learning outcomes. The inclusion of retention intervals aims to eliminate transient effects resulting from practice, such as fatigue or motivational factors [32]. Existing research lacks discussion on how to determine the length of the retention interval. In this study, the retention interval was determined using a combination of experience, design of relevant literature, and course scheduling. One month after the end of training is the latest time the participants can schedule a test before entering the semester vacation. One year after the end of training is the latest time the participants can schedule a test before graduation. Both time nodes are supported by relevant literature studies [33-35]. Within 1 year after completing the training, participants participate in a uniform dental laboratory internship, and the internship outline has uniform requirements for the design of RPDs with the same workload. At the same time, the user accounts of the participants were blocked in the RPD module, preventing the participants from using the module for additional training. However, participants may use paper and pencil for additional practice since they have different expectations for work content after graduation. Therefore, confounding factors related to different amounts of practice are inevitable.

We noted that the scores obtained on the test after 1 month for cohort 1 was significantly lower than the scores for cohorts 2 and 3, who received the D-RPD intervention. This finding reflects the higher efficacy of the D-RPD digital training approach compared to traditional training at improving short-term performance in RPD design. In addition, cohort 2 scored less than cohort 3, which was provided with the 3D virtual cast–based progressive intervention, albeit with no statistically significant difference, which is consistent with the results of Mahrous et al [30]. This finding suggests no significant short-term benefits of progressive digital training incorporating 3D virtual simulation over digital training using 2D sketches and text alone. However, the scores obtained in the tests conducted 1 year later showed that cohort 3 displayed significantly improved performance in comparison with the other cohorts, thus demonstrating improved long-term outcomes of the progressive digital training approach. Of note, added tacit knowledge from clinical practice gained during the internship curriculum that commenced soon after the first test, where students had additional opportunities to learn and participate in the process of RPD design, could have contributed to such an effect. Such practice enriches the experiential learning of students by allowing for case-based learning and greater practice [36]. The D-RPD module with the 3D virtual simulation–based intervention for cohort 3 was aligned with routine clinical production models to a large extent, which possibly facilitated higher competency over a period of time in cohort 3. These findings are in contrast to our short-term observations and those of Mahrous et al [30]. For the “time spent” evaluation dimension, the differences between the 3 cohorts were not significant at the 1-month test. The complexity of the RPD design process itself could account for this finding. After 1 year, a significant difference was notable, and cohort 3 showed the longest mean time spent on the test. It is feasible that the participants in cohort 3 took more factors into account in the RPD design after undertaking clinical practice in the intervening period and that the D-RPD process with 3D virtual casts was the most consistent with clinical practice; therefore, this effect was produced over a longer period of time. The longer time taken by cohort 3 in estimating more factors and spending more time could also have contributed to their higher score over time. These results indicate that the use of D-RPD, especially when incorporating the use of 3D virtual casts in a progressive mode, may facilitate an improvement in the RPD design competency of students compared with the traditional RPD design training approach.

The mean scores of cohorts 1 and 2 were less after 1 year compared to the scores after 1 month, showing a certain degree of loss of competency over time. In contrast, a significant increase was noted in the mean score of cohort 3. Before entering clinical practice, the participants in this cohort were exposed to experiential learning through virtual simulation that had similarities with clinical work, which might have produced a synergistic effect on improving RPD design competency. It is especially noteworthy that the scores and time spent at the 2 time points by cohort 1 showed very large SDs, indicating high variability in RPD design competency among the cohort 1 students. The opposite was notable in cohorts 2 and 3, which may be related to better teacher supervision, which D-RPD can facilitate. Moreover, previous research has shown that D-RPD design training has advantages that can be partly attributed to improved tracking of students’ learning progress and their timely interactions with trainers [37,38]. D-RPD allows teachers to check the progress of the RPD design tasks of the students, make efficient corrections, and provide more frequent feedback. It is evident that this digital teaching mode can facilitate greater student engagement and problem-based learning compared to traditional paper-based teaching. These findings also reinforce that the approach involving D-RPD design combined with 3D virtual casts can provide students with more effective teacher supervision, while offering them virtual experiential learning consistent with clinical activity.

The intervention mode for cohort 3 was similar to the clinical CAD/CAM digital denture design process, which can improve the quality and efficiency of prosthesis design and facilitate improved management of design schemes [39,40]. Intraoral scanning produces 3D virtual casts that can improve precision, and it is readily accepted by the patient compared to the traditional impression method, producing models with higher accuracy [41,42]. The digital workflow allows dental technicians to design directly on these models and to perform postprocessing of multiple scanning data [14,43], thus rendering the entire workflow efficient and convenient. However, despite the rapidly increasing adoption of digital workflows in dental practices worldwide, preclinical education in dentistry and dental technology is typically lagging at imparting the relevant skills to students [12,44,45]. Taken together with our earlier research, this work proposes and validates a progressive digital teaching module for RPD design training that incorporates 3D virtual simulation, demonstrates greater efficacy for a digital training approach compared to traditional training, and provides evidence that a virtual 3D simulation–based progressive digital training module can enhance long-term learning outcomes of RPD design training.

### Limitations and Future Work

The limitations of this study include a small sample size, a single center for recruitment, and a lack of randomization, which may have led to unaccounted differences in the inherent learning ability of students and their existing competency prior to participation in the experiment. In future work, bias may be avoided by using a randomized controlled study design to provide stronger evidence for this training module. In addition, there is a lack of data regarding the effectiveness of this training module for clinical dentistry students. Further studies are merited to enable more widespread adoption of 3D virtual simulation–based digital training approaches in dental education.

### Conclusions

In this cohort study, we report in detail a major update to the D-RPD module and the design of an intervention experiment to observe the effects of traditional training, D-RPD training, and additional 3D virtual simulation–based digital training on the RPD design competency of students. Based on the results, we propose an effective, progressive, digital 3D virtual simulation workflow–based training module for RPD design, and we have preliminarily verified the efficacy of this novel training approach for facilitating improvement and long-term retention of RPD design competency among dental technology students. This training module should be further extended to clinical dentistry students, randomized controlled experiments should be designed, and feedback from students and teachers should be collected to enable its further optimization and eventual inclusion in curricula.

## Supplementary material

10.2196/46789 Multimedia Appendix 1 Development of a progressive D-RPD module incorporating case-based virtual 3D simulation.

10.2196/46789 Multimedia Appendix 2 Display of the interventions of the three cohorts.

## Abbreviations

CAD: computer-aided design
CAM: computer-aided manufacturing
D-RPD: digital removable partial denture
OMEDT: Objective Manipulative Skill Examination of Dental Technicians
RPD: removable partial denture
STROBE: Strengthening the Reporting of Observational Studies in Epidemiology
