# Correction: Efficacy of a Virtual 3D Simulation–Based Digital Training Module for Building Dental Technology Students’ Long-Term Competency in Removable Partial Denture Design: Prospective Cohort Study

**DOI:** 10.2196/63113

**Published:** 2024-08-19

**Authors:** KeXin Liu, YaQian Xu, ChaoYi Ma, Na Yu, FaBing Tan, Yi Li, YaXin Bai, XiaoMing Fu, JiaWu Wan, DongQi Fan, HuBin Yin, MeiXi Chen, HongJi Chen, Lin Jiang, JinLin Song, Ping Ji, XiaoHan Zhao, MengWei Pang

**Affiliations:** 1 College of Stomatology, Chongqing Medical University, Chongqing, China; 2 Chongqing Key Laboratory of Oral Diseases and Biomedical Sciences, Chongqing, China; 3 Chongqing Municipal Key Laboratory of Oral Biomedical Engineering of Higher Education, Chongqing, China; 4 Beijing Unidraw Virtual Reality Technology Research Institute Co, Ltd, Beijing, China; 5 State Key Laboratory of Virtual Reality Technology and Systems, BeiHang University, Beijing, China

In “Efficacy of a Virtual 3D Simulation–Based Digital Training Module for Building Dental Technology Students’ Long-Term Competency in Removable Partial Denture Design: Prospective Cohort Study” (JMIR Serious Games 2024;12:e46789) the authors made one addition.

The “Acknowledgments” section has been amended within the manuscript to read as follows:

KXL and YQX are co-first authors of this work, and XHZ and MWP are co-corresponding authors (XHZ can be reached at haoxiaohan_buaa@163.com for correspondence).

The engineers’ group from Beijing Unidraw Virtual Reality Technology Research Institute Co. Ltd., led by XZ, helped develop the Objective Manipulative Skill Examination of Dental Technicians system, including the digital removable partial denture module. The Zhaozhi student team assisted in the early design and pre-experimental data collection for the digital removable partial denture module. This research was funded by the CQMU Program for Youth Innovation in Future Medicine (W0002 for LJ), and the Chongqing Municipal Teaching Reformation Fund (193070 for XF).

The correction will appear in the online version of the paper on the JMIR Publications website on August 19, 2024, together with the publication of this correction notice. Because this was made after submission to PubMed, PubMed Central, and other full-text repositories, the corrected article has also been resubmitted to those repositories.

